# Recombinant antibody mixtures; optimization of cell line generation and single-batch manufacturing processes

**DOI:** 10.1186/1753-6561-5-S8-O2

**Published:** 2011-11-22

**Authors:** Søren K Rasmussen, Lars S Nielsen, Christian Müller, Thomas Bouquin, Henrik Næsted, Nina T Mønster, Frank Nygaard, Dietmar Weilguny, Torben P Frandsen, Anne B Tolstrup

**Affiliations:** 1 Symphogen A/S, Elektrovej 375, 2800 Lyngby, Denmark

## Background

Recombinant antibody mixtures represent an important new class of antibody therapeutics as demonstrated by the increasing amount of literature showing that combinations of two or more antibodies show superiority compared to monoclonal antibodies (mAbs) for treatment of cancer and infectious diseases [1-5]. Sym004, composed of two antibodies targeting non-overlapping epitopes of the epidermal growth factor receptor (EGFR) act in a synergistic manner to induce an efficient internalization of EGFR leading to subsequent degradation and exhibit superior anticancer efficacy as demonstrated in several preclinical *in vivo* models [5].

At Symphogen A/S, we have developed an expression platform, Sympress™, for cost-efficient production of antibody mixtures. The antibody mixtures are produced using a single-batch manufacturing approach where a polyclonal working cell bank (pWCB) prepared by mixing the individual stable cell lines producing all the desired antibodies is used as seed material for a bioreactor process [6]. By using a single-batch approach the CMC development costs of antibody mixtures are comparable to costs for monoclonal antibodies. However, the single-batch manufacturing approach raises questions with regard to control of composition ratios, compositional stability and robustness of the cell banking procedure.

Here, we present experimental data addressing these key questions and demonstrate that mixtures of recombinant antibodies can be produced under predictable, reproducible and stable conditions using the Sympress™ technology.

## Material and methods

The second generation Sympress technology is based on expression in the ECHO cell line, a genetically modified version of the dihydrofolate reductase (DHFR) negative Chinese Hamster Ovary (CHO) cell line DG44 [7].

The expression plasmid used for stable transfection contained a bidirectional CMV promoter construct enabling co-expression of the IgG light and heavy chains from one plasmid. A DHFR selection marker was coupled directly to the heavy chain via an internal ribosome entry site (IRES).

ECHO parental cells were transfected separately with each of the individual antibody expression vectors using standard transfection technology, whereafter cells were subjected to a methotrexate (MTX) selection schedule. The selected stable pools were single-cell cloned by FACS and high-expressing clones were adapted to chemically defined cell culture medium, expanded and frozen, still as individual monoclonal cell lines.

The preparation of polyclonal cell banks followed a two-tiered cell banking approach. First, the relevant monoclonal cell lines were thawed and expanded. They were then mixed in predefined ratios and frozen as polyclonal master cell banks (pMCB). The pMCB was subsequently thawed, expanded and frozen as polyclonal working cell bank (pWCB).

Antibody purification was performed by capture on a MabSelect SuRe column and cation exchange chromatography (CIEX) was used to separate the different antibodies based on their charge differences. The characteristics of the chromatograms (peak area/peak height) were used to determine the relative distribution of the different antibodies in the mixtures.

## Results

**Figure 1:**
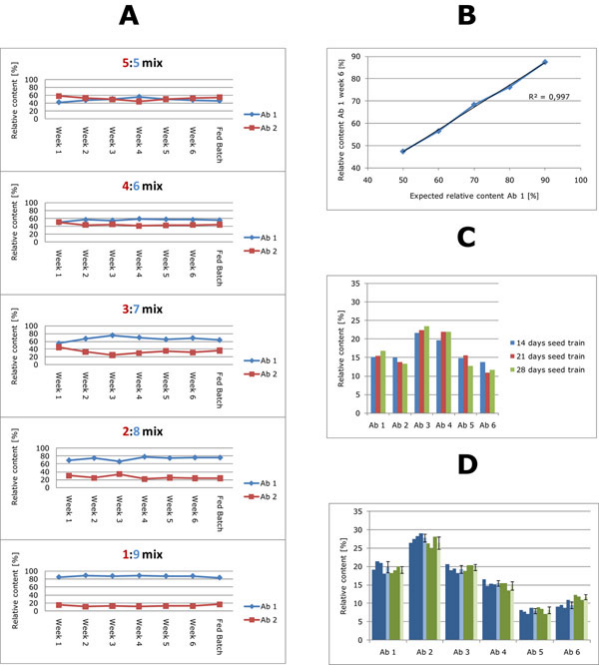
A: Relative antibody ratios in pMCBs generated by mixing two ECHO cell lines at five different ratios. The antibody ratios were determined once a week by CIEX (from week 1 to 6 of cultivation) and in the harvest of a 14-day fed batch cultivation initiated after 6 weeks of cultivation. Red lines represent antibody 1 (Ab 1) and blue lines represent antibody 2 (Ab 2). B: Expected percentages plotted against measured percentages of Ab 1 in the five different mixes after 6 weeks seed train. C: Antibody compositions in pWCB in fed batch shaker experiments after 14, 21 and 28 days seed train, respectively. D:Antibody distributions in harvest from seven different 5 L bioreactor processes. Four pWCB-1 ampoules (blue columns) and three pWCB-2 ampoules (green columns) were used for seed train. The lighter colored columns represent mean ± sdev.

Two ECHO cell lines producing two distinct antibodies were selected based on their growth and production characteristics for a study to examine how mixing of the cells at different ratios affected the antibody composition. Briefly, the two cell lines were thawed, expanded and mixed in five different rations (5:5; 4:6; 3:7; 2:8 and 1:9). The resulting mixtures were frozen as pMCBs. To examine the obtained ratios and the compositional stability over prolonged periods of cultivation the pMCBs were revived and subjected to six weeks cultivation in shakers, followed by a 14 days fed batch process in shakers. Supernatant samples were harvested once a week and at the end of the fed batch process. IgG was captured by protein A and the relative antibody ratio was determined by CIEX. The results clearly showed that all the compositions were very stable over time and, importantly, that the relative amount of each antibody could be controlled by mixing the cell lines in an appropriate ratio (Figure 1A). Furthermore, the was a very strong correlation (R2 = 0.997) between expected and measured percentage of Ab 1 as shown in figure 1B.

We then examined the compositional stability in a more complex model composed of a mixture of six different antibodies. A seed train of approximately 3 weeks would be required to generate cells for inoculation of production reactors in a 10.000 L scaled-up manufacturing process. To examine the robustness of the relative cell compositions during this timeframe +/- one week the cells from pWCB were inoculated into a 14 days fed batch shaker after 14, 21 or 28 days of expansion, respectively. The antibody distributions at the three different time points were very constant as illustrated in Figure 1C. Additionally, the growth and production properties were also very constant over time (data not shown).

The reproducibility and robustness of the cell banking system was evaluated by comparing the antibody product composition produced from two different pWCBs generated from the same pMCB. A pMCB producing a mixture containing 6 antibodies was used for the study. We generated two polyclonal working cell banks (pWCB-1 and pWCB-2) from the same pMCB. Production of pWCB-2 was separated in time from production of pWCB-1 by several months. Four ampoules from pWCB-1 were expanded and used for inoculation of four 5 L bioreactors for fed batch production. Several months later, three ampoules from pWCB-2 were expanded and used to perform three new 5 L bioreactor runs. The antibodies were purified and antibody distribution determined by CIEX. The observed variation, both within the same pWCB and between different pWCB in regard to the antibody distributing was very limited (Figure 1D). This strongly indication that the pMCB/pWCB concept provides a reliable cell banking strategy. The reproducibility was also confirmed with respect to cell growth and productivity (data not shown).

## Conclusion

Symphogen A/S has developed an expression platform, Sympress™, that can be used for predictable, reproducible, and stable production of antibody mixtures in a cost effective setting.

Here, we have shown that the relative antibody ratio in antibody mixtures can be effective controlled using appropriate mixing of the individual monoclonal cell lines before generation of the pMCB. Further, we have presented consistent data from fed batch productions with pWCB where the seed train lengths varied from 14 to 28 days. This strongly supports that the single-batch manufacturing concept is sufficiently robust for manufacturing, also at scales required for commercial manufacturing. The two-tiered cell bank approach composed of pMCB and pWCB is robust and reproducible, and showed very high consistency both within a given pWCB and between different polyclonal working cell banks generated from the same pMCB.
